# Frontotemporal Dementia & the Criminal Legal System: Care Partner Stories

**DOI:** 10.1093/geroni/igaf122.1036

**Published:** 2025-12-31

**Authors:** Victoria Helmly

**Affiliations:** Georgia State University, Atlanta, Georgia, United States

## Abstract

There is limited research on the connection between dementia and the criminal legal system, though we know that this system encounters people with dementia regularly. Prior research has established that individuals affected by frontotemporal dementia (FTD) may have symptoms, including behavioral symptoms, which violate social norms and may be considered “criminal.” As a result, persons living with FTD may be more vulnerable and at risk for interactions with the criminal legal system, including with police. This session will present findings from a study investigating the experiences of people with FTD who have had contact with the criminal legal system through arrest, detention, arraignment, or incarceration through the perspective of their care partners. Preliminary analysis reveals that this group has diverse experiences and perspectives depending upon their individual circumstances. Despite this heterogeneity, they face common barriers, mostly due to challenges in receiving a correct diagnosis and the criminal legal system’s lack of awareness and education about FTD. Care partners rely on shared strengths such as education, social capital, and advocacy skills to navigate these challenges effectively.

